# Genetics of cerebral malaria: pathogenesis, biomarkers and emerging therapeutic interventions

**DOI:** 10.1186/s13578-022-00830-6

**Published:** 2022-06-17

**Authors:** Lydia Nkuah Nortey, Alberta Serwah Anning, Gideon Kwesi Nakotey, Abdala Mumuni Ussif, Yeboah Kwaku Opoku, Silas Acheampong Osei, Benjamin Aboagye, George Ghartey-Kwansah

**Affiliations:** 1 Department of Biomedical Sciences, School of Allied Health Sciences, College of Health and Allied Sciences, University of Cape Coast, Cape Coast, Ghana; 2 Department of Forensic Sciences, School of Biological Sciences, College of Agriculture and Natural Sciences, University of Cape Coast, Cape Coast, Ghana; 3 Department of Biology Education, Faculty of Science, University of Education, Winneba, Ghana; 4 School of Pharmacy and Pharmaceutical Sciences, College of Health and Allied Sciences, University of Cape Coast, Cape Coast, Ghana

**Keywords:** Blood–brain barrier, Cerebral malaria, Clumping, Infected erythrocyte membrane, Mortality, Rosetting, PfEMP-1, Var gene

## Abstract

**Background:**

Cerebral malaria (CM) is a preeminent cause of severe disease and premature deaths in Sub-Saharan Africa, where an estimated 90% of cases occur. The key features of CM are a deep, unarousable coma that persists for longer than 1 h in patients with peripheral *Plasmodium falciparum* and no other explanation for encephalopathy. Significant research efforts on CM in the last few decades have focused on unravelling the molecular underpinnings of the disease pathogenesis and the identification of potential targets for therapeutic or pharmacologic intervention. These efforts have been greatly aided by the generation and study of mouse models of CM, which have provided great insights into key events of CM pathogenesis, revealed an interesting interplay of host versus parasite factors that determine the progression of malaria to severe disease and exposed possible targets for therapeutic intervention in severe disease.

**Main Body:**

This paper reviews our current understanding of the pathogenic and immunologic factors involved in CM. We present the current view of the roles of certain gene products e.g., the var gene, ABCA-1, ICAM-1, TNF-alpha, CD-36, PfEMP-1 and G6PD, in CM pathogenesis. We also present alterations in the blood–brain barrier as a consequence of disease proliferation as well as complicated host and parasite interactions, including the T-cell immune reaction, reduced deformation of erythrocytes and cytoadherence. We further looked at recent advances in cerebral malaria treatment interventions by emphasizing on biomarkers, new diagnostic tools and emerging therapeutic options.

**Conclusion:**

Finally, we discuss how the current understanding of some of these pathogenic and immunologic factors could inform the development of novel therapeutic interventions to fight CM.

## Background

Malaria constitutes a significant and urgent threat to public health in sub-Saharan Africa, where more than 90% of cases occur. In 2018 alone, an estimated 213 million cases of malaria and more than 380 000 deaths due to malaria occurred in this region [1]. The high malaria burden in endemic countries over the millennia has placed great selective pressure on the genome, which has resulted in the selection and expansion of gene variants that protect against severe disease and death. The most important of these known gene variants that confer such protection against malaria are summarized in Table 1. Current efforts to understand and control malaria have centred on understanding the nature of these variants and the manner of the protection they confer, with the view to exploit the defensive mechanisms inherent in these gene variants for the development of novel therapies [2].

**Table 1:** A description and main sources of the malaria defense gene variants analyzed

Gene	Converted protein	Variations	Mechanism	Sources
HBB	β-globin	Sickle hemoglobin heterozygous carriers (HbAS)	Acquired host immunity and improved phagocytosis of the ring-parasitized variant RBC’s Cytoadherence and resetting. Disrupted RBC trafficking of parasite proteins. Suppression of the parasite development owing to oxygen-dependent sickle hemoglobin polymerization	[158–160]
HBB	beta-globin	Heterozygosity of Beta thalassemia	Enhanced antibody binding and subsequent clearance of infected variant RBCs Increased phagocytosis of ring-parasitized variant RBCs	[161]
HBA	α-globin	α-thalassemia (removal of one or more normal 4 genes of α-globin)	Increased phagocytosis of infected variant RBCs by monocytes Enhanced antibody binding and subsequent clearance of infected variant RBCs	[162–165]
G6PD	Glucose-6-phosphate dehydrogenase (G6PD)	G6PD deficiency (G6PDd) for female heterozygotes	Increased ring parasite phagocytosis of red blood cell because of increased oxidative pressure	[2, 166]
CR-1	Complement Receptor 1	Polymorphism of Swain Langley two (Sl2)	Decreased Sl2 RBC attachment to PfEMP1 rosetting parasite binding site	[167, 168]
FY	Chemokines Duffy antigen receptor (DARC)	FY*ES allele	Suppression of Duffy's negative RBC P. vivax invasion by impairment of junction formation	[169, 170]
ABO	Glycosyltransferase enzyme	Blood group O deletion of single nucleotide ABO (rs8176719)	Reduced *P. falciparum* rosetting	[2, 75, 171]
ATP2B4	PMCA4 calcium transporter	ATP2B4 polymorphisms of single nucleotides (rs4951074 and rs1541255)	Changed transcription factors bound to enhancer components of ATP2B4, owing to reduced expression of genes, followed by a disorderly intracellular calcium homeostasis	[75, 172]
GYP	Glycophorins	Making copies of the Dantu blood group GYPB-A encoded by the hybrid genes	Inactivation of the invasion of *P. falciparum* because of excessive Dantu RBC membrane pressure	[173–175]
IL12-RB2	Complex receptors for Interleukin 23 and 12	Clusters of IL23R-IL12RB2 single nucleotide	Immunomodulatory functions in malaria defensive immunity	[176, 177]

Malaria is caused by *Plasmodium* parasites of which five different species are known to cause human disease: *P. falciparum*, *P. vivax*, *P. malariae*, *P. ovale* and *P. knowlesi* [3]. Of the five species of *Plasmodium sp* known to cause human disease, *P. falciparum* causes by far the most severe malaria, although serious disease and death due to *P. vivax* and *P. knowlesi* also exist [4, 5]. Specifically, severe disease and death are commonly associated with *P. falciparum* and *P. vivax,* owing at least in part, to their ability to produce high-density parasite infections [6–8]. The life cycle of *Plasmodium sp* has been described extensively in the literature [9–11]. In brief, the cycle begins when *Plasmodium* parasites, known at this stage as sporozoites, enter a human or other suitable vertebrate host, usually through the bite of female *Anopheles* mosquitoes. Sporozoites migrate to the liver, where they infect hepatocytes and, through an asexual reproductive process called schizogony, produce large numbers of “daughter” parasites called merozoites that are released into the blood. Merozoites invade erythrocytes and undergo further cycles of schizogony to produce more merozoites. The cycle in the human or vertebrate host ends when some merozoites develop into male and female gametocytes, which constitute the sexual development phase of *Plasmodium.*

The cycle within the *Anopheles* vector begins when gametocytes are ingested in a blood meal from an infected vertebrate host. The gametes fuse within the gut of the mosquito to produce a diploid zygote that undergoes meiosis and differentiation to form a motile ookinete. The ookinete penetrates the midgut and forms an oocyst beneath the gut epithelium. Several cycles of mitosis within the oocyst called sporogony result in the production of numerous sporozoites. A mature oocyst ruptures and releases the sporozoites into the hemolymph, which migrate to the salivary glands of the vector, ready to infect another vertebrate host when the vector feeds. The entire developmental cycle lasts 10–18 days. The clinical sequelae of *Plasmodium* inoculation can range from asymptomatic parasitaemia through uncomplicated illness to severe malaria and death. [5].

### Cerebral Malaria (CM)

Severe malaria, including CM, commonly develops in infected children in the first few years of life or migrants from non-endemic regions of the world [12]. This observation, however, is reflective of the protective role of prior malaria episodes and have been reported to be associated with the upregulation of components of both the innate and adaptive immune systems [13, 14]. Additionally, an increasing number of gene variants that confer specific immunity to malaria in endemic regions of the world (Table 1) exists, which significantly modify malaria severity and fatality. For decades, a massive international effort coordinated by the World Health Organization (WHO) has laboured with some success to reduce malaria morbidity and mortality. The latest effort dubbed the Global Malaria control Program (GMP) has chalked significant successes in decreasing malaria morbidity and mortality since its adoption in 2015 [15]. Nevertheless in the last decade, the proportion of *Plasmodium falciparum*-infected patients who died from severe malaria (SM) complicated with CM mortality ratio between 0.15 and 0.25 in children—remains relatively unchanged [16]. Ergo, reducing the case incidence of CM could significantly cut malaria deaths. But this in turn depends on deciphering the molecular events that define the transition from asymptomatic parasitaemia and uncomplicated malaria to severe manifestations of the disease. Decades of sustained research on CM pathology have revealed some of the multifactorial influences that lead to the main post-mortem findings in CM patients [17–20] which underlies the impaired consciousness and coma in CM patients [21]. A new Magnetic resonance imaging (MRI) research has found that swelling of the brain is highly related to lethal outcomes in pediatric cases of CM [22]. This review examines the current understanding of the roles played by key genes and gene products in various pathologic and immune processes in CM, as well as the immune responses and biochemical changes provoked by parasitaemia, which may be beneficial or harmful to the recovery process.

### Clinical features of cerebral malaria

**Fig. 1:**
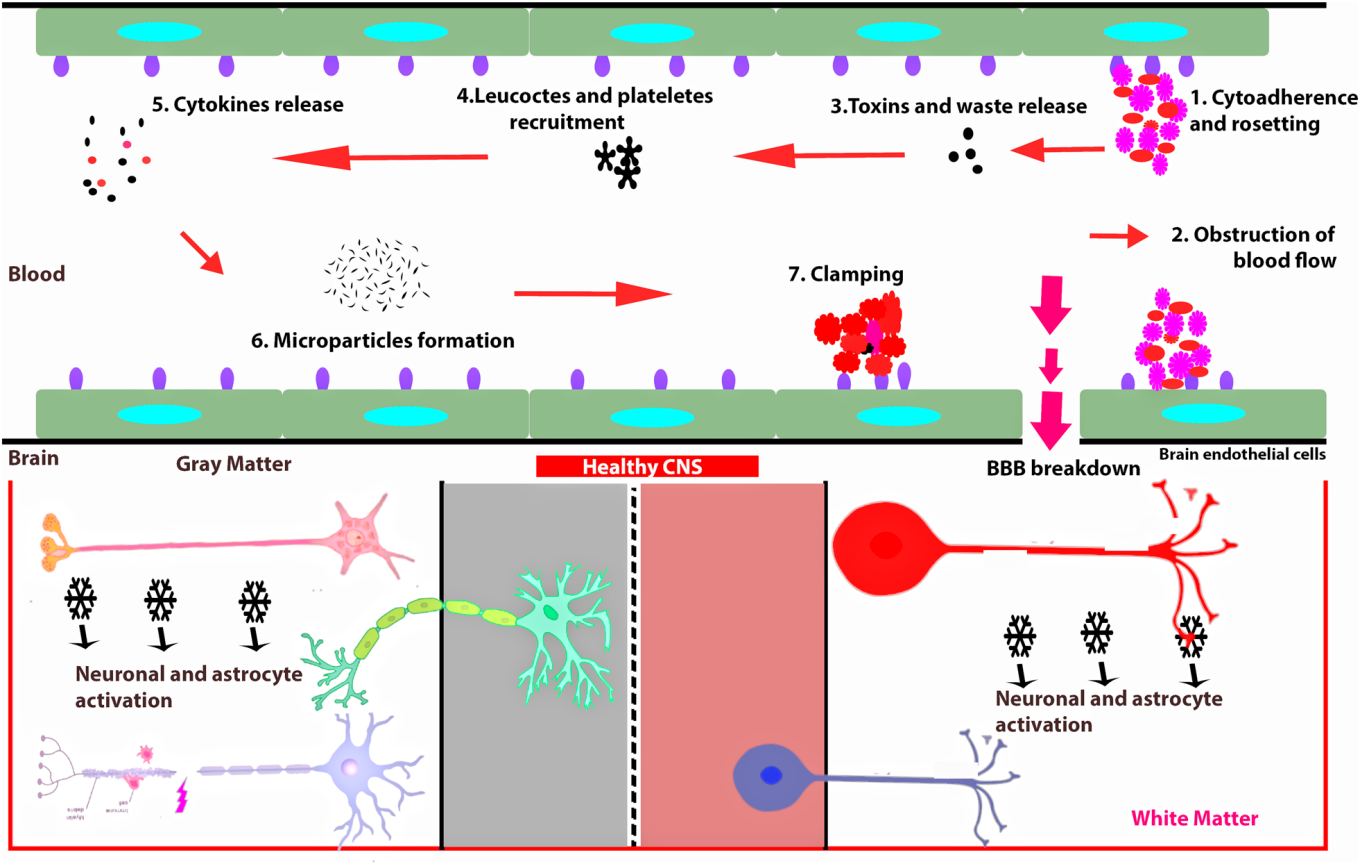
Schematic abstract of the pathology of cerebral malaria. In both white and gray content, disease occurs differently. Although hemorrhagic spots abound in white matter, its presence in the gray matter remains unclear. Within these brain zones, the blood vessels of the brain are unique and can lead to PRBC driven by var gene expression, differential attachment of PfEMP1 and the resulting stimulation of opposite signaling pathways in the brain endothelial vascular system. Astroglial stimulation is enabled by the release of cytokines and chemokines into the brain from the irritated endothelial cells of BBB in connection with its activation to both neurotoxic plasma agents and dissolved *Plasmodium* triggers. This causes brain injury, together with the inflow of immune cells which contributes to the neurological effect after the CM

*P. falciparum* infection has been the most important neurological problem with the psychological condition of distinctive consciousness being the severe form of CM. Pediatric malaria-like CM has a reappearing diurnal fever developed after parasite release when parasitized red blood cells (PRBC) rupture, second to asexual reproduction and secretion of cytokines [23]. In CM, a rapid, permanent unconscious state, and/or convulsion may occur in patients with acute infection without returning to consciousness. Other indicators include hepatic and spleen enlargement, jaundice, lung oedema, kidney disease, pallor, hypoglycemia, haemorrhage, hypertension and severe anaemia [24]. Focal neural symptoms may also exist in some cases [25]. Because age is a key determinant for CM, variations between that of adults and children have been thoroughly assessed [26]. Children increasingly exhibit symptoms of brainstem dysfunction at the end stages of the disorder. This may lead to deficit in sensory and motor function, cranial nerve palsies, impairment of consciousness, dysautonomia, and respiratory failure [27]. These clinical signs and symptoms are often linked with other encephalopathies and the endemic ones sometimes harbour subclinical parasites [28]. Malignant retinopathy characterizes CM [27] and this occurrence represents a brain pathogenic process comprising cerebral parasite sequestration [29]. The confirmation of malarial retinopathy is the best way to distinguish malarial from a non-malarial induced coma. Malarial retinopathy diagnosis consists of four main components: retinal whitening, vessel changes, retinal haemorrhage, and papilledema. The first two of these abnormalities are specific to malaria but absent from other ocular or systemic conditions [30]. Retinopathy, however, is a condition that can affect both children and adults alike [31]. Interestingly, autopsies of some individuals with CM have revealed no indication of parasitized Red Blood Cells (PRBCs) sequestration in the cerebrovascular system. Retinopathy-free CM might be the result of an asymptomatic accidental parasitemia followed by a second (perhaps viral) exposure that causes fever and coma. This idea has been validated by clinical autopsy investigations which discovered alternative infectious and non-infectious causes of coma in individuals dying of retinopathy-negative CM [32]. Other autopsy findings revealed that isolation of PRBCs in circulation entails axonal disruption, myelin degradation, and a disintegrated blood–brain barrier (BBB) [33] as shown in Fig. 1.

Neurological problems in survivors of CM are prevalent in African children with rates in retinopathy-positive individuals surpassing 30%. Differences in cognitive and behavioural aspects, motor abnormalities, and even epilepsy are all manifestations of these impairments. The most common symptoms are temporal, cognitive, and motor problems, followed by disruptive behavioural disorders, and eventually epilepsy [34]. Many CM survivors suffer from long-term brain damage, as well as behavioural and mental health issues (common in children) [35]. Adults with hemiplegia, cerebral palsy, cortical blindness, deafness, cerebellar ataxia, and impaired cognition and learning are among the long-term consequences of CM [36].

### Pathogenesis of cerebral malaria

According to WHO, Cerebral *P. falciparum* malaria (CM) is defined as a deep, unstable coma that lasts for over 1 h after a seizure in a patient with peripheral *P. falciparum* parasitemia without any trigger for encephalopathy regardless of anticonvulsant medications [37]. The etiology of CM is most likely a multi-factorial process, with sequestration, inflammation, and endothelial dysfunction in the microvasculature of the brain being the most significant contributors to coma induction in individuals with severe malaria. It is best described by parasite sequestration, which results in PRBC engorgement of cerebral capillaries and post-capillary venules, triggering an inflammatory cytokine response and vascular leakage [38]. Increased lactate and alanine concentrations in such individuals show that these episodes eventually lead to cerebral hypoxia. However, the sequence of events that initiate these pathophysiological processes, as well as the role that their intricate interplay plays in the development of CM, are largely unclear [39].

The host receptor *Plasmodium falciparum* erythrocyte membrane protein 1-Domain cassette 8 (PfEMP1-DC8) has been discovered as an endothelial cell protein C receptor (EPCR) on brain endothelial cells which interact with PRBC [40], as well as intercellular adhesion molecule-1 (ICAM-1), which has been demonstrated to be associated with CM development. Once connected to EPCR, PRBCs prevent the transformation of inactive protein C to activated protein C, a thrombin-inhibitor that may contribute to localized endothelial activation. The PRBCs' adherence to endothelium ultimately decreases the level of EPCR connections that are needed to regulate proper homeostasis. Because the brain endothelium has a relatively low level of expression of these receptors compared to other tissues, it is hypothesized that the brain is particularly vulnerable to inflammation due to decreased thrombin production regulation. This indicates a key role in the localized pathophysiology of coagulation/inflammation in CM [41]. This impacts numerous signals, which eventually lead to an increased angiopoietin-2 (Ang-2)/angiopoietin -1, TNF, Von Wilbrand Factor (VWF) production, leading to the loss of endothelial barrier function. This affects the dissemination of thrombin and increases thrombin synthesis [41].

Ang-1 stabilizes the endothelium, whereas Ang-2 increases vascular permeability and regulates different endothelial receptors. Ang-1 and Ang-2 are controlled by the use of nitric oxide (NO) generated as a substratum in the endothelium. NO production leads to vasorelaxation, adhesion molecules downregulation and a decrease in thrombosis [42]. Malaria infection may lower L-arginine levels and result in hypoargininemia that drastically reduces NO bioavailability [43]. The reduction of active NOs in the system might lead to the over-expression of certain endothelium receptors, notably ICAM-1, a well-known PRBC binding receptor [44]. With the increased ICAM-1 presence caused by the decrease of the NO, the sequestration of CM metabolic acidosis and thrombocytopenia may lead to additional sequestration of the PRBC, uninfected RBC’s, leukocytes and activated platelets.

As already stated, malaria infection generally results in an upregulation of VWF in the blood plasma. It is said to be produced by the stimulation of endothelial cells that increases the synthesis of thrombin during infection [45] particularly in CM. This leads to higher cellular excretion of VWF tied to higher circulation in the host. As the extremely big proteins of the VWF are released more often, the platelets might be sequestrated, the PRBCs recruited and endothelial cell permeability altered, resulting in microvascular thrombosis. In addition to the overexpression of VWF, several cytokines and chemokines have been consistently linked to CM severity. PRBCs eventually stimulate an immunological response in the circulation and rupture of PRBC's and increase the local production of inflammatory cytokines such as TNF-α. Increased amounts of these inflammatory mediators could be responsible for increased leukocyte sequestration in the brain microvasculature during CM. The increase in leucocyte sequestration is expected to promote local inflammation, increase endothelial reception expression and result in the disposal of soluble endothelial receptors, leading to possible endoscopic injury [39].

Also, microvascular flow is eventually reduced due to the cytoadherence of the PRBCs and unparasitized RBCs towards endothelial receptors. This obstacle in arterial flow reduces normal oxygen and glucose supply to critical organs and tissues. Brain microvasculature impediment is a mechanism believed to contribute to comas induction in persons with CM [46]. In addition to the above-mentioned concept on microvasculature blockage, a number of additional processes are also suggested but the specific causes of coma remain elusive. Furthermore, elevated tau proteins levels have demonstrated pathological changes of neuronal disruption in the cerebrospinal fluid of children with CM. The above statements affirm BBB's major function in CM, as it is located at the PRBC interface intravascularly and neuronally dangerous (Fig. 1). The PfEMP-1 encodes together with the var gene and communicates with numerous host receptors, for example, ICAM-1, EPCR, CD36 etc. [47, 48], depending on which var gene is expressed. PRBC binding PfEMP-1 differential encoding of the var genes to each of the receptors leads to host signals downstream, including inflammatory and coagulatory pathways activation, which leads ultimately to the BBB disintegration including encephalopathy deficiency. Moreover, in vitro studies with cerebral endothelial cells [49], indicate the varying endothelial vulnerability of the individual influence the development of CM. This is demonstrated by significant vesicle nuclei, endothelial degradation of cell binding molecular substances (ICAM-1, VCAM-1, E-sectin), the release of cytokines, the dissolution of a BBB [50] and the turn-on of NF-kB transcription factors. CM endothelial disruption is shown in vitro [51], as well as in vivo in both human CM and murine models [52]. Studies of Murine CM have demonstrated, through releases of Granzym B and/or perforin [53] that these T cells that further transmigrate into neuropil damages the neural cells. It is not currently known whether CD8+ T cells preferably infiltrate those regions of the brain of white matter or gray matter. Further pathways for neuronal damage in selected neurons, as seen in human CM, can include the use of caspases [54, 55]. Sphingolipid changes can also affect vascular integrity and lymphocyte transmigration. For example, a reduction in brain trafficking and reduced peripheral IFNβ levels in therapeutic blocking agents like FTY720 has been revealed. However, not all tests detected a connection of peripheral cytokines with cerebral oedema [56]. In addition to increased coagulation factors and changes in blood metabolites, sequestration and inflammation are both associated with CM neuropathogenesis, which can emerge in specific areas in the white or gray matter [57–61].

**Fig. 2:**
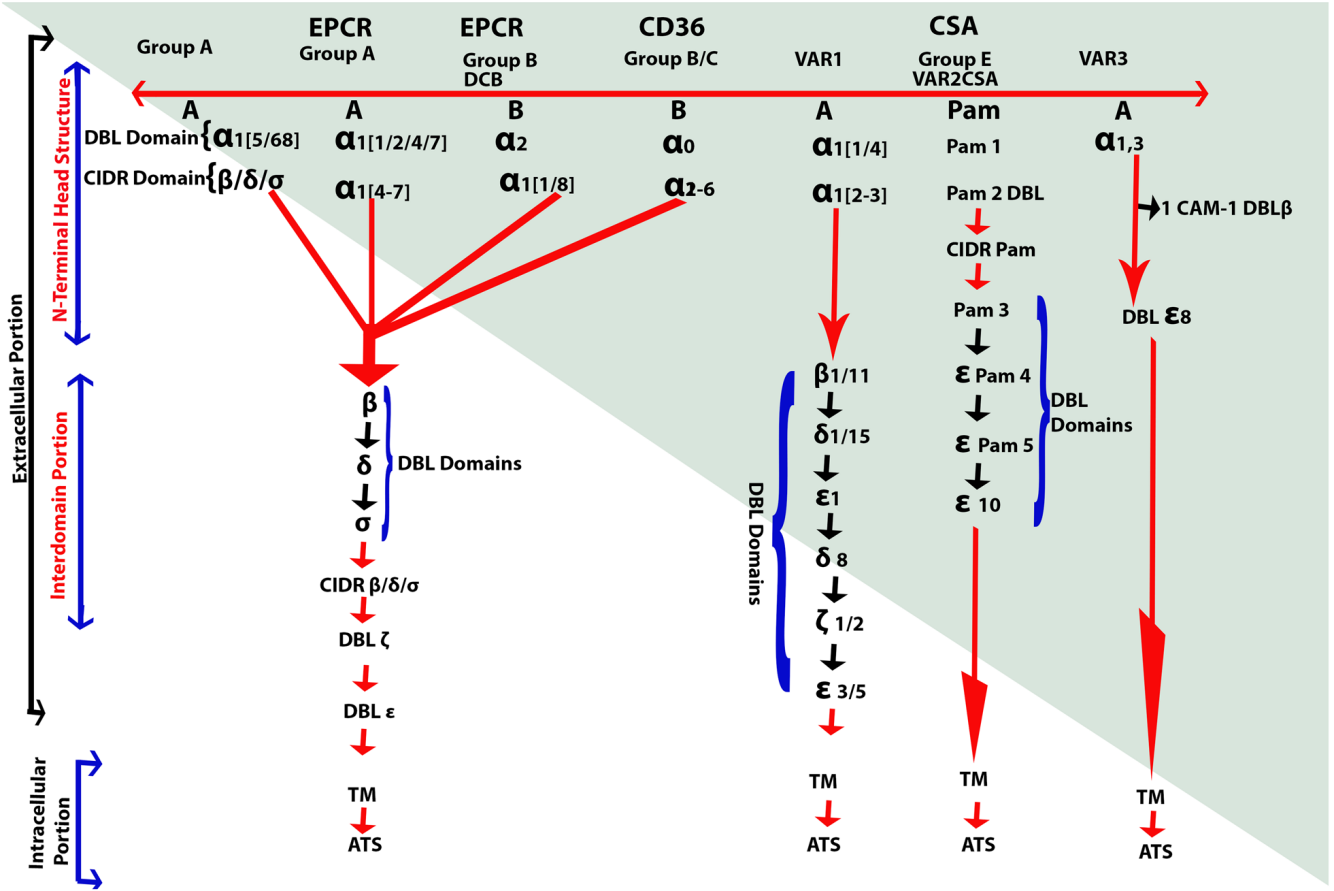
PfEMP1 domain structure: the diagram shows the PfEMP1 domain structure consisting of the N-terminal head-structure, the C-terminal domains 2–6, the Domain (TM) and the ATS region, with defined bold-receptors. Receptor properties are determined by the combined CIDR and DBL domains and by separate CIDRa head arrangement domains that have mutually exclusive to EPCR and CD36 binding. Section A PfEMP1 and part B/A chimeric subset PfEMP1 (DC8) connect to EP CRI through CIDRa1, although CD36 is linked with CIDRa2-6 by Group B and Group C PfEMP1.Placental chondroitin sulfate A (CSA) binds via the DBLpam1, DBLpam2 and E atypical VAR2CSA PfEMP1 group. The VAR 1, VAR3 and CIDRb/c/d domains group A binding phenotype is surprising, although they neither bind the EPCR nor the CD36. DBLb domains from both classes A and B may be active in ICAM-1 binding. The other DBL domains (c/d/e/f) are not much understood, but IgM and a2-macroglobulins are involved with DBLe and DBLf domains

*P. falciparum* erythrocytes membrane protein 1 (PfEMP1) is the primary mediator of endothelium parasite cytoadherence. This is a modification of the surface antigen expressed [47, 62] on the infected erythrocyte (IE) surface on the body coded with about 60 var genes for a parasite genome [47]. The PfEMP1 consists of a series of Duffy binding-like (DBL) and Cysteine-rich (CIDR) domain areas, characterized into four major classes (A, B, C and E) under the 50 Upstream-Var gene-encoding sequence (Fig. 2 [63]). ICAM 1 is also mediated via DBLb domains adjacent to CIDR domains, with a dual binding phenotype combined with that of the EPCR in some cases [64]. *P. falciparum* parasites, as described above, leave behind PfEMP1 molecules on the surface of the erythrocytes that they invade. These high molecular weight variant proteins effectively mediate adherence to IEs to a variety of host receivers [64], following a 16-h post-invasion accounting for the presence of only young ring stage IEs in the extravascular. IE binding induced by PfEMP1 to particular receptors is a critical indicator for clinical effects of *P. falciparum* infections in main tissues and organs. This theory has been proven when placental malarial diseases are induced by VAR2CSA (a highly polymorphic multidomain protein, usually consisting of six DBL domains) type PfEMP1 in placental affinity for chondroitin sulfate A (CSA).[65]. In addition, the defensive immunity to placental malaria is well known in relation to the acquisition of IgG and VAR2CSA expression in CSA adhesion. Such results raised the expectations that in other types of serious *P. falciparum*, not at least CM [66], other unique PfEMP1 and host receptors may play similar and decisively significant roles.

**Fig. 3:**
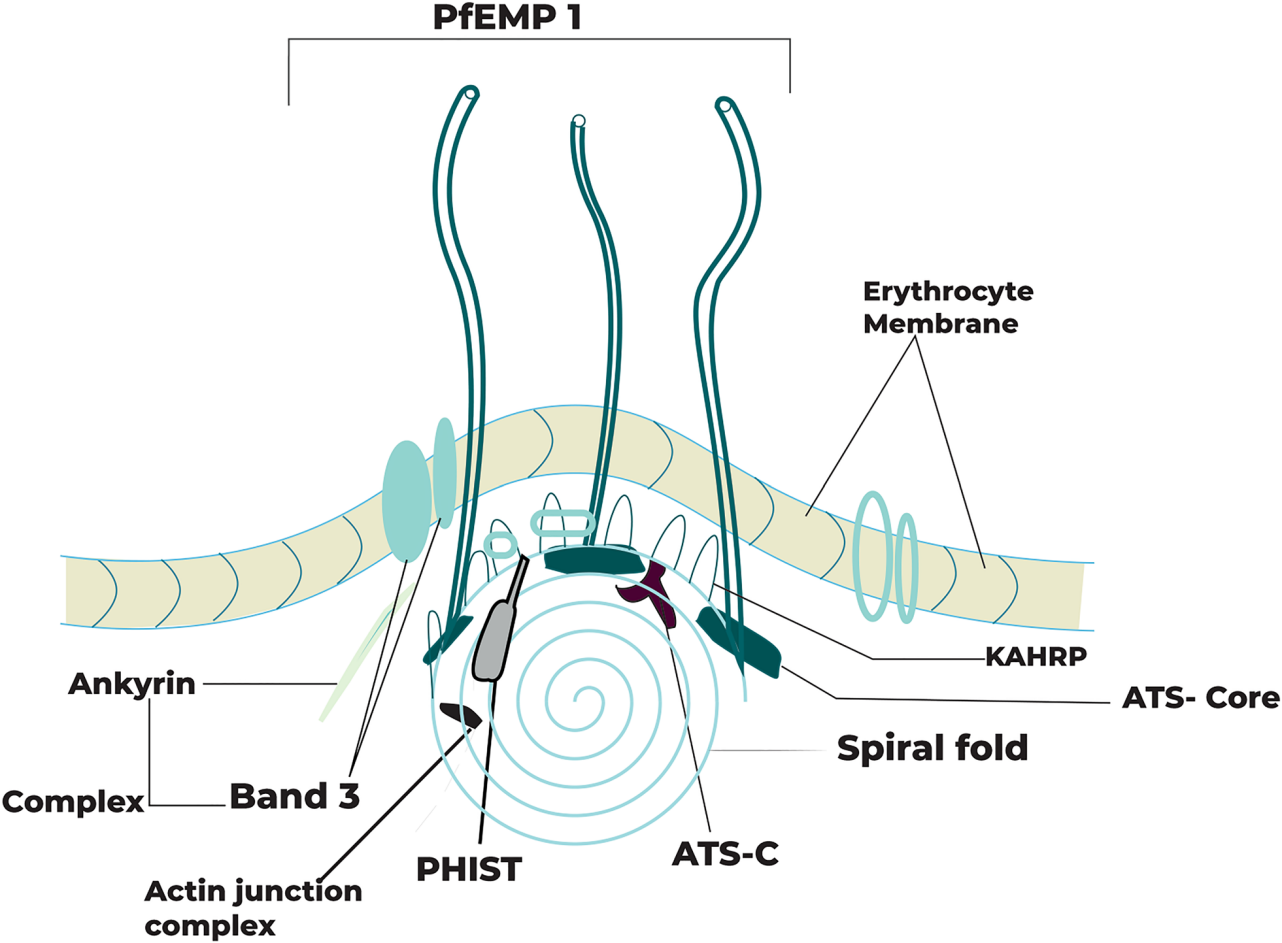
Presentation of PfEMP1 on IE surface knobs: the base of the knob complex is KAHRP. Averagely, at the end of every switch, there are three PfEMP1 molecules. KAHRP binding to the spectrum is essential in the spiral structure but KAHRP alone does not seem to belong to the spiral

PfEMP1 is limited to membrane knobs on IE surface [17]. With the inclusion of PfEMP1, knobs develop together with host and parasite molecules, such as parasite-encoded knob-based proteins rich in histidine (KAHRP). This multiply into a 5-molecule spiral-cone structure associated with erythrocyte cytoskeletal complexes of spectrin-anchyrine as shown in Fig. 3 [18].

### The Barrier of Blood–Brain (BBB)

The blood–brain barrier serves as the connection between the blood and brain parenchyma needed to sustain a semipermeable and strong cerebral homeostasis for transcellular molecular bidirectional transportation (from glucose, amino acid, transferrin, activated plasma protein etc.). Parts of the BBB are microvascular endothelial cells that form a permanent barrier through strong linkages (tight junctions), a basement membrane, pericytes as well as astrocytes in immediate communication including nerve cells and microglia. Endothelial brain cells differ from endothelial cells of other bodies both structurally and functionally [67]. In specific, they have close intercellular connections and conformity, which usually inhibit small and wide molecular paracellular diffusion and prevent blood cell penetration to the brain parenchyma. BBB disorder is a neurological disorder [68] that is considered a characteristic of CM [69]. It is triggered by inflammation of the brain endothelial by the aggregation and retrieval of IEs and leukocytes including platelets [70].

These findings collaborate observed reduced endothelial tolerance to trans modification in the gene regulation of these brain endothelial cell junctions as seen in in vitro studies [71]. The permeability of the BBB can be affected by various parasites and host factors involved in CM pathology. Among these aspects is a number of other causes [72], including hemozoin-induced matrix metalloproteases (MMP) and cytokines (IL-1α, IL1β, IL-6, TNFα). For example, MMP is engineered to provide tight attachment proteins (ZO-1, ZO-02 claudine-5), believed to cause serious deterioration of the junction, enhanced parcel leakage causing ischemic and inflammatory insult to the BBB gap. Once the BBB is split up, astrocytes and neurons may be stimulated by leukocytes, cytokines, chemokines and soluble parasites into the brain parenchyma, leading to coma damage [73].

### Genetic tolerance and vulnerability of the host

Malaria is a genetic source of inherited erythrocyte diseases including sickle cell, thalassemia and Glucose-6 dehydrogenase deficient disorders, as seen in Table 1 above. In Africa alone, more than 1 million children died of *P. falciparum* a year before the twenty-first century [20]. Indicators of inflammation observed in countries like Nigeria, Malawi and Mali with IL 17, type 1 interferon receptor varieties (IF1) [19], and IL4 & IL22, respectively are leading host genetic factors to vulnerability to CM. [74]. Furthermore, previous studies found that CM [33] has a role in intercellular molecular adhesion-1 (ICAM-1) varieties. Lately, a trial has been performed within Kilifi, Kenya, where15 genetic molecules have been identified for increased pediatric malaria [75]. Again in an Indian trial, TNF polymorphisms have been identified [76]. Furthermore, epidemiological studies have documented the correlation of malaria outcome with age and previous epigenetic exposure [77–79]. There is also increasing evidence that repeated parasite infections can contribute to gene expression variations in malaria-like phenotypic traits tolerance using a Toll-like receptor excitable effect (TLR) [33]. These epigenetic changes have in fact been documented among Kenyan infected children with *Plasmodium* [77]. Co-infections of pediatric patients with CM, such as HIV, are known to have different mortality risk factors. Autopsy tests have shown that the number of intravascular monocytes in HIV-infected children who died of CM has increased [53]. For humans with CM co-infected with HIV, an increased presence of T cells was also found [80, 81]. It is possible that the pathologic damage to the CM, resulting in an elevated T-cell inflow into the brain is further amplified by the immune dysregulation in coinfected patients. [81, 82]. Taken together, different host factors lead to the vulnerability of extreme malaria and although regions vary, good host-immune response factors appear essential.

### Parasitized erythrocytes isolation in the brain

In the post-capillary venules in certain organs, like the lungs, stomach, intestines, brain and placenta, IEs bind to endothelial host receptors [83]. This isolation induces circulatory system diseases and inflammation, and individual multi-organ dysfunctions including renal, liver, and placental impairment in CM [84, 85]. There is therefore long-lasting important priority research to classify *P. falciparum* binding sites, especially of the specific variants of PFEMP1 which direct IE tissue isolation with the receptor of the host they cleave to. ICAM1 (CD54) was early identified as an endothelial IE receptor and known for a long time to be of interest to specific aggregation of IEs in the brain of CM patients. In addition, it has been documented that African CM children are selectively bound to ICAM1 in vitro by isolated *falciparum* parasites [63]. Even so, there are also records of the contrary and isolates from Asian adult malaria patients seeming not to display preferential conformity to ICAM-1. Eventually, several experiments struggled to find proof of elevated levels of ICAM-1 expression in the brains of lethal CM victims. IE adherence in ICAM 1 can also be regulated by the variants of the PfEMP1, which can also link with either the EPCR or the CD (Fig. 2). The DC4 family was initially identified by a quest for orthology of the pfd1235w gene in patients with Ghanaian parasites based on the relationship of PFD1235w with severe malaria [48, 86, 87]. The search culminated in the creation of a panel of pfd1235w genetically different parasites linking ICAM1 by DBLβ4 domain PfEMP1, presented on the IE surface [66]. The C terminal of ICAM1-binding Motif was shown to be a sequence for these domains. The causes also found in some PfEMP1 proteins of Group A outside of DC4 (incl. certain types DC5 and DC13), and in some variants of group B/A [64], are limited to the DBLβ domains directly downward of the DC15 and DC13-binding C domains of the EPCR-binding subtype [88]. The cognate receptor for the DC8 or DC13 PfEMP1 domain cassette proteins in Group A is the C cognitive protein recipient. DC8 PfEMP1 is located in Group B PfEMP1, which evolved from the combination of ancestral Group A and B var genes. DC13 PfEMP1 variants are common and attach voraciously to endothelial pulmonary, cardiac and bone-marrow cells [89]. DC8 proteins containing PfEMP1 may be one of those initially expressed in infections in early life that have adhesion properties that provide a survival benefit to IEs among malaria-naive children. In addition, *P. falciparum* Parasites also transcribe DC8 and DC13 encoding var genes at a high level in African children and Indian adults with severe malaria, including CM [90, 91]. Analysis indicates further the importance to CM pathogenesis by observing the choice of IEs for adherence to the endothelial brain cells for the use of certain domains [91]. Eventually, EPCR binding PfEMP1 variants of Group A have been associated with brain swelling [48] which is a significant fatal contributor in pediatric CM [86]. A study of Kenyan childhood with CM, for example, found no evidence that would support specific enrichment to DC8- or DC13-compatible PfEMP1 variants for children with retinopathy. Not all PfEMP1 variants that can bind ICAM1 are also EPCR-binding, as stated before. In fact, only one [92] of the DBLβ ICAM-01 domains detected prior to the establishment of DC4 in Group A were identified for Group B Protein and Group C Protein. Such PfEMP1 proteins, as all containing CD36-binding CIDRα domains upstream of the ICAM-1-bound DBLβ domain, seem to be in binary selection for adhesion to ICAM-1 and CD36. This is expected because CD36 is a normally binds to IE and most non-placental P adhesive receptor. CD36 is the affinity of PfEMP1 protein [93], but is not present in Groups A and PfEMP1 B/A influencing extreme disease and also not in persons with reduced immunity to malaria. Instead, CD36 attachment is linked with uncomplicated malaria [94], and seems to have been formed to promote IE sequestration in tissues other than the cortex, where CD36 is missing or only sparkling. In accordance, the adhesion phenotype with CM is IEs with PfEMP1 group A proteins (including DC4) that make concurrent ICAM1 and EPCR binding (‘double binders’) [64]. Eventually, while many other molecules were involved as IE adhesion receptors, including those molecules which tend to be expressed on the endothelium of the brain, none were relevant to the seriousness of the disease.

### Rosetting and clumping of infected erythrocytes

Not only is the endothelium adhered to by IEs, but also by uninfected erythrocytes. Initial reports indicate rosetting in fragile monkeys infected with *Plasmodium*. [95]. Rosettes were documented to form from infected erythrocytes isolated from patients with CM at slightly higher levels than IEs in patients with uncomplicated malaria [96]. Findings show that the occlusion of brain microvessels is mainly caused by IEs, although, uninfected erythrocytes contribute to the rosetting. For instance, IEs that sequester in microvasculature expresses different variants of PfEMP1 which also binds to the micro-vessels of the brain and other shared receptors of erythrocytes and PfEMP1 enabling separate endothelial and erythrocyte receptors to also bind concurrently. Unsurprisingly, several studies have shown significant associations between rosetting and the severity of malaria/CM [97]. Even though the explanation for this divergence is not settled entirely, it may represent real geographical disparities in Southeast Asia where the relation is not normally found and Africa where the relation is found. For the other receptor-specific adhesive IE phenotypes and malaria seriousness, similar geographical differences have been described. If these geographical variations do indeed occur, variations will occur among parasites, hosts and/or intensity of transfer. These factors remain entirely undefined in terms of proportional contributions. Conversely, whether PfEMP1-dependent rosetting occurs or not is determined by the receptor specificity of the PfEMP1 variant expressed. Soluble plasma factors may be of pathological interest as a result of PfEMP1 binding. Thus, IE in vitro rosetting usually (but not always) entails the involvement of plasma or serum in the test and many factors have been involved. The most studied is IgM, which may clearly boost the formation of rosettes [98]. Their function is independent of antimicrobial specificity since it is mediated by Fc instead of Fab. Moreover, pentameric verification of IgM [99] is possibly needed since IgM enhances rosette formation by bridging multiple PfEMP1 molecules and boosts their collective avidity for the erythrocyte receptor [100].

## Cerebral malaria treatment interventions

In recent years, advanced imaging techniques have been more widely available in malaria-endemic countries, allowing for a leap forward in clinical research aiming at unraveling the etiology of CM.

Magnetic resonance imaging (MRI) gives a measure of various neurological characteristics that are hard to examine otherwise, such as the type of cerebral blood flow change and damage to brain tissue. The study by Penet et al. was the first to use MRI to examine vascular damage in a mouse model of CM, and they discovered inflammatory-related vascular damage [101, 102]. MRI methods were employed subsequently to investigate neuronal axon damage in 120 Malawian children with CM, including scattered cerebral edema of the brainstem. [103]. The use of MRI has been important in revealing an elevated intracranial pressure and brain stem herniation in fatal instances by enabling the comparison of particular parameters between CM patients living and those who fell victim to the disease [27, 104, 105].

Unlike MRI, computer tomography (CT) scans are now commonly available in malaria-endemic countries. Their pioneering use in CM patients from Thailand and Kenya suggested for the first time the involvement of cerebral edema in the development of the pathology [106]. Subsequently, a study in India showed that CT findings correlate well with level of consciousness and severity of disease but do not reveal the extent of the pathology permitted by post-mortem examinations [107]. CT imaging is particularly helpful for the determination of cerebral volume variation and the detection of infarctions in large vessels, as demonstrated in a pediatric CM patient population [108].

### Investigative neuro-imaging tools

While the use of neuroimaging techniques has contributed to a better understanding of the pathophysiology of CM, novel and revolutionary approaches have become available in the laboratory but are not applicable directly to patients. For this, the experimental model, albeit limited [109], represents a useful tool to investigate the pathogenesis of CM at the cellular and molecular level in the brain.

In vivo bioluminescent imaging is a versatile and sensitive tool that is based on the detection of light emission from cells or tissues. The technique has been allowed by the genetic modification of malaria parasites and the production of luciferase-expressing lines. This, coupled with the development of imaging systems to detect cells expressing reporter genes, has significantly broadened the possibilities for in vivo studies of interactions between *Plasmodium* spp. parasites and their hosts [110]. Optical imaging by bioluminescence allows a low-cost, non-invasive and real-time analysis of disease processes at the molecular level in experimental animals. It also permits longitudinal monitoring of the course of the pathology in the same animal, and the imaging of transgenic fluorescent or bioluminescent malaria parasites is now widely used as a tool to assess parasite distribution during experimental CM. A recent study used real-time in vivo imaging to evaluate the contribution of different immune mediators to PRBC accumulation and distribution during the development of experimental CM. The results showed that CD8+ T cells and IFN-γ are responsible for the rapid increase in total parasite biomass, as well as for the accumulation of PRBC in the brain and in different organs [111]. These in vivo pathogenesis studies can also be carried out with a different bioluminescent target, as shown by Imai and colleagues, who evaluated oxidative stress during experimental CM using OKD48 (Keap1-dependent Oxidative stress Detector, No-48-luciferase) mice. Oxidative stress in the brain can be visualized in these animals after injection of luciferin, and an elevated bioluminescent signal was associated with the development of the pathology [112]. Lastly, this imaging technique can also be used for the assessment of parasite virulence [113] and provides a simple and reliable framework for in vivo antimalarial and CM adjunctive treatment screening by monitoring post-treatment changes in bioluminescence signal, which correlates with the degree of parasitemia in the animal [112].

Recently developed Intra-vital microscopy, is an advanced imaging tool that allows the direct and live visualization of the brain via a cranial opening [114]. The technique can reveal cellular responses over time and space during the course of experimental CM and can be conducted under conditions closely approximating those of a natural environment. In addition, it presents the advantage of observing in vivo pathological events in the brain, including variations in hemodynamic events and vascular leakages [115]. By comparing the variation of these parameters between control and treated animal groups, intra-vital microscopy has allowed the assessment of intervention drugs, including nimodipine and nitric oxide (NO) therapy [116]. It is a versatile platform, as demonstrated by two recent and innovative studies. First, Cabrales and colleagues performed the direct, quantitative, and dynamic analysis of fluctuations of oxygen transport and tension during experimental CM progression and its contribution to the severity of disease. Results highlighted the pial tissue as highly sensitive to changes in blood flow, anemia, and low oxygen tension impacting sufficient oxygen delivery [117]. Second, Pai and colleagues used of a novel, two photon-based approach, which allowed them to monitor the behavior of leukocytes in cerebral microvessels during the development of the pathology in infected mice. A decrease in the rolling velocity of monocytes, a measure of endothelial cell activation, was associated with the progressive worsening of signs in the animals. These modifications were mediated by *Plasmodium*-specific CD8+ T lymphocytes, suggesting their direct influence in the regulation of vascular pathology associated with the development of experimental CM [118].

The 18F-fluorodeoxyglucose (FDG) positron emission tomography (PET) is a non-invasive imaging tool used to map cerebral metabolic activity by quantifying the uptake of a glucose analogue by brain cells. This metabolic activity was measured in vivo during the progression of experimental CM in the *Plasmodium coatneyi* primate model of the pathology. The analysis revealed diffuse and heterogeneous reduction of metabolism in the cortex during the acute phase of infection [112]. These results are consistent with a focal impairment of the microcirculation, potentially induced by PRBC sequestration. However, it is plausible that this reduced metabolic activity safeguards the cerebral tissue against hypoperfusion, as an inherent function of the microcirculatory system is to protect organs from the effects of diminished oxygen and metabolite supply. This could explain why more than half of CM patients present no neurological sequelae following recovery [119]. Another study showed reduced cerebral blood flow during CM using an FDG-PET in a murine model of experimental CM. FDG-PET was recently used systematically in a cohort of patients to help the diagnosis of fever of unknown origin [120], showing that this approach might become available to CM patients soon and may be able to complement the ongoing MRI studies to shed some light on the pathophysiological processes during the neurological syndrome.

### New diagnostic tools

In addition to prevention strategies and effective treatment, one of the most important factors influencing the outcome of CM is its early diagnosis. According to a study published in 2004, about a quarter of the pediatric patients diagnosed with CM using the WHO criteria were shown at autopsy to have died of non-CM causes [121], which highlights the importance of accurate and reliable diagnostic tools.

### Malarial retinopathy

The sequestration of *P. falciparum*-infected red blood cells (PRBC) in the cerebral microvasculature is the hallmark of CM. In pediatric patients, retinal microvessels have been shown to sustain damage comparable to the ones occurring in the brain, making them an easily observable surrogate marker to assess the severity of cerebral pathology during CM [122]. In recent years, the approaches adopted to assess and document the retinal changes during CM have evolved rapidly and are now available for clinical studies in endemic areas.

Fundoscopy is a relatively low-cost and easy technique to assess the presence of retinopathies, which allows the accurate distinction between malaria and non-malaria coma in CM patients. Retinal changes include vessel color changes, white-centered hemorrhages, and peri- and extramacular whitening. The severity of these changes during *P. falciparum* infection correlates strongly with patient mortality, and the identification of markers associated with the presence of retinopathies and therefore, of CM, may allow the early detection of patients at risk [123].

Optical coherence tomography (OCT) is an in vivo imaging tool for the detection of retinal changes. This imaging technique allows optical-signal acquisition by which high-resolution cross-sectional images of the retina, optic nerve-head and even the thickness of the retinal nerve fibre layer can be acquired and both qualitative and quantitative evaluations can be made [124]. Despite its non-invasive nature and high-resolution output, the use of OCT in malarial retinopathy has been difficult to implement systematically so far due to its costs, as well as practical issues. Indeed, patients need to be seated upright for the retinal analysis, which is problematic for comatose CM patients in intensive care units. However, a case of P. vivax retinopathy has been recently described using OCT [125], showing that the new development in high-resolution and high-speed OCT, along with the improvement in portability [126, 127] might make the technology extremely valuable for retinopathy analyses in malaria-endemic areas.

While the use of retinopathy has helped increasing the accuracy of diagnosis in African children and more recently in Asian adults [128], its use is still infrequent, as systematic fundoscopy requires a trained ophthalmologist, as well as expensive equipment that is not always available in field clinics. This has led to the recent development of easy-to-handle and affordable retinal cameras [129], as well as the emergence of modified handheld portable devices such as smartphones. In addition to their telecommunication functions, the most recent models possess diagnostic-quality imaging facility that meet the criteria necessary for accurate fundus examination and rapid diagnosis of retinopathy [130]. This revolutionary “teleophthalmology” can be performed using cheap 3D printed fittings where the built-in flash of the phone provides the light source, and an installed application allows the easy and rapid photo-documentation of retinal abnormalities in CM patients [131]. Such devices can be operated by healthcare workers after minimal training and the saved images can be sent by SMS or email to an ophthalmologist for rapid diagnosis.

In recent years, the use of Electroencephalography (EEG) has allowed the detection of these delayed CM sequelae, including neurodisabilities such as status epilepticus. Acute and serial EEGs are especially important for identifying subclinical seizures. A study performed in Kenya revealed that in about 25% of the enrolled pediatric CM patients, coma is due to continuing subtle seizure activity which is likely to go undetected, but is responsive to anticonvulsant drugs [132]. Subsequent studies in Kenya and in Mali showed an increased prevalence of epilepsy in patients who survived CM. The recent inclusion of retinopathy as a [133] criteria for CM diagnostic in a study in Malawian children helped to improve the accuracy of the diagnosis in enrolled patients, and to identify children with pre-existing neurological injuries, predispositions to adverse neurological outcomes, or non-malarial causes of coma. In this carefully defined cohort, almost a third of retinopathy-positive CM survivors developed epilepsy or other neurobehavioral sequelae [134].

One major limitation of the EEG studies is the serial post-discharge follow-up assessments, which involve multiple patient visits to the hospital, or home-based visits by nurses. These are not always possible and often present a logistical hindrance to EEG studies in the field. However, a miniature version of the EEG equipment, known as Micro-EEG, is now available as a portable headgear that can accommodate up to 32 electrodes and connects via Bluetooth technology to a small monitoring machine. The micro-EEG diagnostic accuracy of status epilepticus is comparable to that of standard EEG systems [135] and will greatly facilitate not only the recording of brain as an easy diagnostic tool [136], but will also allow an easier continuous recording of the patient after discharge.

### Biomarkers for cerebral malaria

Biomarkers include tools and technologies that can facilitate the prediction, cause, diagnosis, progression, regression, or outcome of treatment of disease. For pathologies of the nervous system, there is a wide range of techniques used to gain information about the brain in both the healthy and diseased state [137]. These involve measurements directly on biological media such as blood or cerebrospinal fluid (CSF) from the patients; or measurements via brain imaging, which do not involve direct sampling of tissue but measure changes in the composition or function of the nervous system [138]. Based on their function and stage(s) of usage, these can be classified as early screening and diagnosis biomarkers, as well as prognostic biomarkers.

### Early screening and diagnosis biomarkers

Biomarkers used for early screening or diagnosis are used as an indicator of a biological factor that represents either a subclinical manifestation, stage of the disorder, or a surrogate manifestation of the disease. Early screening biomarkers allow the identification of individuals destined to become affected or who are in the “preclinical” stages of the illness [139]. Unfortunately, due to the rapid development of CM and the late presentation of patients to hospitals, longitudinal analyses of plasma from patients with falciparum malaria have not been feasible so far thereby hampering the potential identification of early screening biomarkers for CM before the onset of symptoms. However, serological factors that allow the accurate discrimination of CM after the onset of symptoms have been described in the recent years. These biomarkers are indicative of pathology, as they are based on specific processes that have been associated with the development of CM [112].

For instance, the role of endothelial intra-cellular adhesion molecule-1 (ICAM-1) in the sequestration of PRBC is well-understood, and specific binding of PRBC to ICAM-1 has been implicated in the development of CM. High levels of plasma soluble ICAM-1 were found to be associated with the development of CM in Ghanaian children, and these levels may reflect the upregulation of ICAM-1 in the cerebral microvasculature [140]. Angiopoetin-1 and -2 (ANG-1 and -2) are critical regulators of endothelial activation and integrity, and their levels have also been described as reliable biomarkers of CM. Indeed, ANG-1 and -2 levels profiled from serum or whole blood were shown to discriminate accurately between cerebral and uncomplicated malaria in African patients, and between cerebral, severe non-cerebral malaria, and uncomplicated malaria in a cohort of Thai patients [141]. Compared to UM, CM patients presented significant decreases in ANG-1 and increases in ANG-2 levels and the ratio of ANG-2: ANG-1. This is consistent with the pathophysiology of CM, which involves endothelial activation and dysfunction. Indeed, ANG-1 maintains vascular quiescence, while ANG-2 displaces ANG-1 upon endothelial activation and sensitizes the cells to become responsive to sub-threshold concentrations of tumor necrosis factor [142]. Estimation of *Plasmodium falciparum* histidine rich protein 2 (PfHRP2) in the plasma samples has also been shown to be an accurate diagnostic tool to ascertain the parasite biomass in severe malaria patients, and allowed the distinction between severe and uncomplicated malaria [143, 144]. Clinical studies have also shown that PfHRP2 can be present in the CSF of patients with CM [145]. More recently, the use of advanced affinity-proteomic tools employing a high-throughput platform of specific antibodies for candidate screening has allowed a wider analysis of potential diagnosis biomarkers for the neuropathology.

### Prognostic biomarkers

Prognostic biomarkers provide information on the likely course of the disease in an individual. Plasma levels of ANG-1 and ANG-2 can also predict the clinical outcome of CM, according to studies performed in African children and in Indian adults [146], in which low ANG-1 levels on presentation was associated with a fatal outcome. This may indicate that, in addition to antiparasitic drugs, ANG-1 is needed to reverse the deleterious endothelial activation in CM and prevent death [147]. CXCL10 and CXCL4 (C-X-C motif chemokine 10 and 4), the ligands of chemokine receptor CXCR3, were described as another set of prognostic biomarkers in CM [148]. Indeed, high plasma levels of the chemokines were found to be significantly associated with mortality in CM patients. CXCL10 is produced by a variety of cells, including endothelial cells. The effects of elevated levels of CXCL10 on the cerebral microvasculature are unknown but are suspected to cause local injuries by recruiting mononuclear leukocytes, inducing focal hyper-inflammation [112]. In addition, CXCL4 is released from activated platelets and stimulates TNF production by mononuclear leukocytes, a key pro-inflammatory cytokine associated with the development of CM. This study established that CXCL10 and CXCL4 can be routinely used to predict the mortality risk in CM patients in endemic settings. Along with the clinical diagnosis of CM, the presence of PfHRP2 in CSF can also be used to predict the disease outcome, as recently demonstrated in retinopathy positive CM patients from Malawi. Using 100 CSF and 103 plasma samples, their findings inferred that an increased level of PfHRP2 in CSF and lower plasma/CSF PfHRP2 ratios was predictive of death in retinopathy positive CM patients [149].

### Future directions and market barriers

Host plasma micro particles (MP) are submicron elements (100 nm–1 μM), which originate from extracellular vesiculation processes during host cell activation and/or apoptotic events. High MP numbers were shown to be significantly higher in the plasma of patients with CM compared to patients with uncomplicated malaria or severe anemia in several separate analyses [150, 151], showing that they could be potentially used as a diagnosis biomarker for CM. In addition, platelet-derived MP were shown to be the most abundant in the plasma of CM patients, and their levels were significantly correlated with coma depth and thrombocytopenia. However, the current analysis of plasma MP necessitate high-sensitivity clinical equipment and trained technicians, which might explain why their use as a biomarker in endemic regions has not been further investigated to date. The fast-paced evolution of low-cost, portable, point-of care quantitative diagnostic devices might reverse the situation in the near future. Similarly, cumulating data suggest that small non-coding-RNAs such as microRNAs (miRNAs) can be utilized as potential biomarkers for the diagnosis and prognosis of a variety of parasitic diseases [152]. Circulating miRNAs can be detected in biological fluids as serum, saliva and others, exhibiting a good potential as non-invasive biomarkers. While this has not yet been evaluated in *falciparum* malaria infection, it is likely that the current technology required for miRNA identification and quantification will restrict their use as diagnostic or prognosis biomarker for now. Lastly, In addition to biomarkers from biological media, the recent implementation of MRI techniques to investigate the factors leading to the development of CM might also lead to the identification of biomarkers of severity and/or disease outcome using imaging maps. Such approaches could focus on the parasite burden in the brain and correlate it with disease severity, or establish a scale of brain swelling in CM patients, potentially indicative of the disease prognosis.

### Emerging therapeutic options

The major challenge to prevent human mortality in CM lies in the current lack of specific therapies aimed at dampening the pro-inflammatory state associated with the neurologic syndrome, as well as its deleterious effects on the host. Considering the multi-factorial nature of the neuropathology, even the most effective anti-malaria drugs cannot ensure complete survival [153] and due to the poor understanding of its pathogenic processes, candidate adjunctive therapies to decrease mortality in CM have been unsuccessful so far [154]. However, the recent breakthrough allowed by some of the technologies and approaches described above are slowly narrowing the spectrum of candidate therapeutic pathways. Some of these novel adjunct therapies include heme-oxygenase-1 (HO-1) and carbon monoxide (CO), exogenous nitric oxide, pressurized oxygen, additive antioxidants, and hydrogen sulfide gas [155].

In addition to adjunct therapies, new classes of agents developed using novel creative strategies are urgently needed to tackle severe malaria infection. Indeed, the number of available and effective antimalarial drugs is quickly dwindling, as the resistance of *P. falciparum* against artemisinin combination treatments (ACT), the recommended first-line therapy for infected patients, is now prevalent across mainland Southeast Asia [156]. This is alarming because first, resistance to the previous mainstays of antimalarial treatments namely chloroquine and sulfadoxine/pyrimethamine have already spread across southeast Asia into Africa, resulting in the deaths of millions of patients [157] and secondly, there are currently no alternative drugs to replace ACT. However, unprecedented global and multidisciplinary efforts aimed at broadening therapeutic potential and identifying novel modes of action are currently in place [157]. Hopefully, these efforts will allow the widening of malaria treatment options and will help to overcome the emerging drug resistance.

## Conclusion

Knowledge on the molecular basis of *P. falciparum* parasites’ relationships with the hosts is advancing rapidly. PfEMP1 is essentially significant for the existence of the parasites making this antigen a key immune target. Furthermore, it has been shown that the gender-specific effect of ACE2 genetic polymorphisms, responsible for the increase of Ang II production has been significantly linked to mild malaria. The development of therapies and vaccines to create a narrowly reactive antibody-response prevention and ideal reversal of IE adherences to ICAM1 and EPCR, which are reported as main receivers in CM pathogenesis will be an obvious aim alongside the existing initiative to improve VAR2CSA-based vaccines. These genetic results can help to understand the pathogenesis of malaria and urge future research among different groups so that these findings can be validated and the genetic significance of these polymorphisms to malaria pathogenesis investigated.

An inhibitory and reverse adhesion of IEs to ICAM 1 is reported in a monoclonal antibody including antibodies that affect “dual binding” variants of PfEMP1. The associated cost of this procedure is, however, likely to prevent its application in clinical practice. Since 2000, incredible levels of intervention coverage in endemic Africa have cut *P. falciparum* infection prevalence in half and clinical illness incidence by 40%. Amidst this encouraging improvement, the numbers remain concerning. The systematic application of some evolving latest techniques targeted at improving the precise diagnosis of malaria-associated complications is likely to assist clinicians identify high-risk CM patients in the future, as well as expedite clinical triage of symptomatic parasitemic patients and their prompt and comprehensive care. This, in effect, should lead to a reduction in malaria-related mortality globally. Furthermore, as demonstrated by a recent and ground-breaking study of brain lesions in Malawian patients with CM, the recent implementation of state-of-the-art investigation tools to elucidate the pathophysiology of CM is likely to result to the detection of new image-derived prognosis biomarkers and adjunct treatment avenues. In summary, a lot has indeed been acquired from decades of CM pathogenesis and immunity research, although many a lot still remains unclear. Such practical studies have offered important insights into the biology of the relationship of host parasites, essential to discovering new approaches to malaria prevention action.

## Data Availability

All data generated or analyzed during this study are included in this published article.

## Abbreviations

ABCA-1: Adenosine Triphosphate Binding Cassette Transporter A1
ACE2: Angiotensin-converting enzyme 2
Ang: Angiotensin
ATS: Acidic Terminal Segment
BBB: Blood Brain Barrier
CD: Cluster of Differentiation
CM: Cerebral Malaria
CIDR: Cysteine Rich Inter-Domain Regions
CIDRa1: Cysteine Rich Inter-Domain Regions Alpha 1
CIDRβ: Cysteine Rich Inter-Domain Regions Beta
CSA: Chondroitin Sulfate A
CSF: Cerebrospinal fluid
CT: Computer Tomography
DBL: Diffuse B-cell Lymphoma
DC: Domain Cassette
EEG: Electroencephalography
EPCR: Endothelial Protein C Receptor
Fc: Fold-change
Fab: Fragment antigen binding
FDG-PET: Fluorodeoxyglucose-Position Emission Tomography
FTY720: Fingolimod
GMP: Guanosine3′,5′-monophosphate
G6PD: Glucose-6-Phosphate Dehydrogenase
G6pdd: Glucose-6-Phosphate Dehydrogenase Deficiency
HIV: Human Immunodeficiency Virus
ICAM-1: Intercellular Adhesion Molecule 1
IE: Infected Erythrocytes
IFNβ: Interferon-beta
IF1: Inhibitory Factor 1
Ig: Immunoglobulin
IL: Interleukin
IL12-RB2: Interleukin 12 receptor, beta 2 subunit
KAHRP: Knob Associated Histidine Rich Protein
MMP: Matrix Metalloproteinases
MP: Micro Particle
MRI: Magnetic Resonance Imaging
NF-kB: Nuclear Factor kappa light chain enhancer of activated B cells
NO: Nitric Oxide
OCT: Optical Coherence Tomography
P: *Plasmodium*
Pfemp-1: *Plasmodium falciparum* erythrocyte membrane protein 1
PRBC: Parasitized Red Blood Cell
RBC: Red blood cell
SM: Severe Malaria
TB: Tuberculosis
TLR: Toll-like receptors
TM: Transmembrane
TNF**-**α: Tumour Necrosis Factor Alpha
VCAM-1: Vascular Cell Adhesion Molecule 1
VWF: Von Willebrand Factor
WHO: World Health Organization
ZO-1: Zonula occludens-1
